# 2-Hydroxyglutarate in Cancer Cells

**DOI:** 10.1089/ars.2019.7902

**Published:** 2020-09-29

**Authors:** Petr Ježek

**Affiliations:** Department of Mitochondrial Physiology, Institute of Physiology of the Czech Academy of Sciences, Prague, Czech Republic.

**Keywords:** 2-hydroxyglutarate, isocitrate dehydrogenase 1 and 2, metabolic reprogramming in cancer, DNA and histone hypermethylation, immune system, tumor cross talk, metabolic marker

## Abstract

***Significance:*** Cancer cells are stabilized in an undifferentiated state similar to stem cells. This leads to profound modifications of their metabolism, which further modifies their genetics and epigenetics as malignancy progresses. Specific metabolites and enzymes may serve as clinical markers of cancer progression.

***Recent Advances:*** Both 2-hydroxyglutarate (2HG) enantiomers are associated with reprogrammed metabolism, in grade III/IV glioma, glioblastoma, and acute myeloid leukemia cells, and numerous other cancer types, while acting also in the cross talk of tumors with immune cells. 2HG contributes to specific alternations in cancer metabolism and developed oxidative stress, while also inducing decisions on the differentiation of naive T lymphocytes, and serves as a signal messenger in immune cells. Moreover, 2HG inhibits chromatin-modifying enzymes, namely 2-oxoglutarate-dependent dioxygenases, and interferes with hypoxia-inducible factor (HIF) transcriptome reprogramming and mammalian target of rapamycin (mTOR) pathway, thus dysregulating gene expression and further promoting cancerogenesis.

***Critical Issues:*** Typically, heterozygous mutations within the active sites of isocitrate dehydrogenase isoform 1 (IDH1)^R132H^ and mitochondrial isocitrate dehydrogenase isoform 2 (IDH2)^R140Q^ provide cells with millimolar r-2-hydroxyglutarate (r-2HG) concentrations, whereas side activities of lactate and malate dehydrogenase form submillimolar s-2-hydroxyglutarate (s-2HG). However, even wild-type IDH1 and IDH2, notably under shifts toward reductive carboxylation glutaminolysis or changes in other enzymes, lead to “intermediate” 0.01–0.1 m*M* 2HG levels, for example, in breast carcinoma compared with 10^−8^ *M* in noncancer cells.

***Future Directions:*** Uncovering further molecular metabolism details specific for given cancer cell types and sequence-specific epigenetic alternations will lead to the design of diagnostic approaches, not only for predicting patients' prognosis or uncovering metastases and tumor remissions but also for early diagnostics.

## Preface

Specific cancer cells, notably grade II/III glioma (35, 36), secondary glioblastoma (127), and acute myeloid leukemia (AML) (16, 60, 103, 144) cells, exhibit heterozygous point mutations in the active sites of cytosolic isocitrate dehydrogenase isoform 1 (IDH1) (8, 11) or isoform 2 (IDH2), localized in the mitochondrial matrix (65, 189). The resulting heterodimeric enzymes play a neomorphic role since they form the oncometabolite r-2-hydroxyglutarate (r-2HG; abbreviated 2HG when enantiomer/stereoisomers are not distinguished) (54, 60, 109, 182). A decade of research has provided a detailed description of the effects of 2HG on the acceleration of oncogenesis, such as affecting epigenetics by hypermethylation *via* inhibitions of 2-oxoglutarate- (2OG-) dependent dioxygenases, blocking DNA and histone demethylation (35, 51, 54, 100, 144). Remarkable details on hypermethylated promoters of certain genes are currently available as well as knowledge of numerous other phenomena, such as interrelationships between tumors and the immune system.

Links have been established between the patient's IDH1 or IDH2 mutation pattern, molecular mechanisms of the alternated epigenetic niche, and reprogrammed metabolism for predicting prognoses for various cancers. The neomorphic activity of mutated IDH1 or IDH2 enzymes causes a dramatic elevation of 2HG levels, which themselves are sufficient to promote gliomagenesis (35) or leukemogenesis in hematopoietic cells through the maintenance of dedifferentiation and increased proliferation (99). Also, a key component of the hypoxia-inducible factor (HIF) pathway, the enzyme prolyl hydroxylase domain-2 (PHD2/EglN1), has been found to be activated by r-2HG (85). Since the oxygen-dependent PHD inhibition initiates HIF-mediated transcriptome reprogramming, also promoting the Warburg glycolytic phenotype, r-2HG should prevent HIF-1α stabilization. However, PHDs are subjected to important HIF-independent fuel-sensing regulations (42).

In contrast, at low concentrations, both 2HG enantiomers participate in not yet fully elucidated metabolic pathways, which may be associated with the regulation of cell proliferation and other functions. A big question is whether “intermediate levels” of 2HG also provide neomorphic effects, such as those naturally produced by nonmutated IDH1 and IDH2; and s-2-hydroxyglutarate (s-2HG) as a by-product of side reactions of other enzymes. Such effects are expected to be weaker and/or slower. For each cancer cell type and/or situation, we should determine to what extent the side formation of 2HG by IDH1/2 and various other enzymes contributes to these “intermediate” levels.

Surprisingly, 2HG can be found in a very wide concentration range. The concentration of 2HG can reach between 1 and ∼30 m*M* in grade II/III gliomas (30, 36, 60), whereas “intermediate levels” of both 2HG enantiomers would be in the 10–100 μ*M* range and their effects in these levels should be further studied. There is no doubt that an imbalance in 2HG formation/degradation very frequently accompanies a specific cancer metabolism. In turn, the reprogrammed metabolism may lead to the further accumulation of 2HG, reaching higher concentrations than in physiological states.

In this review, I briefly discuss the known metabolic pathways involving 2HG, the concomitantly reprogrammed metabolism, oxidative shifts in redox homeostasis, and the effects resulting from 2HG accumulation promoting cancerogenesis, as well as the role of 2HG in interactions of tumors with the immune system.

The main metabolic changes connected to increased 2HG enantiomer levels induce alternations in redox homeostasis, such as decreasing NADPH/NADP^+^ and NADH/NAD^+^ ratios, increased reactive oxygen species (ROS) formation, or decreased antioxidant defense. Possible roles of these states are discussed. The states are not only passive reflections of the altered metabolism but also represent factors that can further accelerate metabolic and other functional or pathological changes. Last but not least, I will attempt to speculate on a possible utilization of 2HG as a prognostic/diagnostic marker, discussing a specific example of breast cancer.

## Metabolism of 2HG

### Isocitrate dehydrogenases IDH1 and IDH2 as sources of r-2HG

#### Canonical reactions of wild-type IDH1 and IDH2

**FIG. 1.**
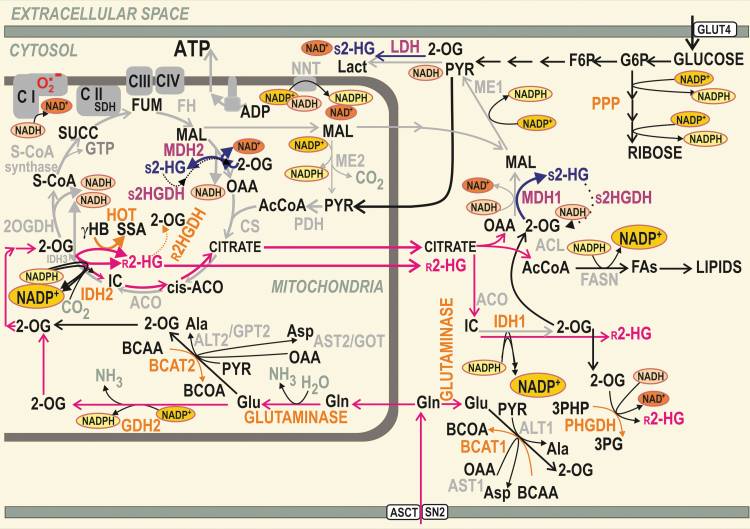
**Typical cancer metabolism related to the formation of r-2HG and s-2HG.** Examples of reactions (not necessary occurring within a single cancer cell type) are depicted within the context of reductive carboxylation reactions of cytosolic IDH1 and mitochondrial IDH2 accompanying glutaminolysis (*neon red arrows*). The concomitant NADPH/NADP^+^ and NADH/NAD^+^ equilibria are emphasized (*larger symbols* point to accumulation of the nicotine amide nucleotide). Complete NADPH-dependent reductive carboxylation by IDH2 leads to IC formation followed by the citrate's export from the mitochondrial matrix. The minor alternative reductive reaction of IDH2 forms r-2HG at the expense of NADPH. HOT/ADHFE1 competes with IDH2 for 2OG, but upon the conversion of γ-hydroxybutyrate (γHB) to SSA also synthesizes r-2HG. Its specific matrix dehydrogenase (r2HGDH) slowly degrades r-2HG, which may be also exported from mitochondria by an as yet unknown mechanism. The minor NADH-dependent side reaction of the matrix malate dehydrogenase MDH2 may also convert 2OG to s-2HG under specific conditions. The resulting s-2HG can be slowly degraded by the s2HGDH. The export of s-2HG is omitted for simplicity. Mitochondrial glutaminase and cancer-specific glutamate dehydrogenase GDH2 (insensitive to GTP inhibition) supply 2OG for IDH2 and HOT reactions and the forward Krebs cycle. 2OG is rather consumed by the branched chain aminotransferase BCAT2 when β-like oxidation of the produced branched-chain oxoacids proceeds. Similar BCAT1-mediated reaction can occur in the cytosol. In contrast, during typical glutaminolysis, 2OG is supplied to the Krebs cycle owing to reactions of alanine aminotransferases ALT2 (also termed glutamate pyruvate transaminase GPT2) or aspartate aminotransferase AST2/GOT2 (glutamate oxaloacetate transaminase 2). In the cytosol, 2OG can be alternatively split into s-2HG by an NADH-dependent side reaction of LDH. 2OG can be similarly converted to s-2HG by the cytosolic MDH1. Also, cytosolic s2HGDH has been described to degrade cytosolic s-2HG. In turn, major cytosolic reactions producing r-2HG are those of IDH1 (NADPH-dependent) and PHGDH (NADH-dependent). γHB, γ-hydroxybutyrate; 2HG, 2-hydroxyglutarate; 2OG, 2-oxoglutarate; ACL, ATP citrate lyase; ACO, aconitase; ASCT, SN2, glutamine carriers; AST2/GOT2, glutamate oxaloacetate transaminase 2; BCAT, BCAA aminotransferases; CS, citrate synthase; FASN, fatty acid synthase; FH, fumarate hydratase; FUM, fumarate; GDH, glutamate dehydrogenase; GLUT, glucose transporter; HOT/ADHFE1, hydroxyacid-oxoacid transhydrogenase/alcohol dehydrogenase iron-dependent isoform 1; IC, isocitrate; IDH1, isocitrate dehydrogenase isoform 1; IDH2, isocitrate dehydrogenase isoform 2; Lact, lactate; LDH, lactate dehydrogenase; MAL, malate; MDH, malate dehydrogenase; OAA, oxaloacetate; PHGDH, phosphoglycerate dehydrogenase; PYR, pyruvate; r-2HG, r-2-hydroxyglutarate; s-2HG, s-2-hydroxyglutarate; S-CoA, succinyl coenzyme A; SDH, succinate dehydrogenase; SSA, succinic semialdehyde; SUCC, succinate.

Cytosolic and peroxisomal IDH1 (94 kDa, EC 1.1.1.41) (111, 190) as well as the mitochondrial isoform IDH2 (94 kDa, EC 1.1.1.42) (22, 89) are homodimeric enzymes, which reversibly catalyze the oxidative decarboxylation of isocitrate (IC) into 2OG and CO_2_, using the cofactors NADP^+^ and Mg^2+^ (133). In contrast, the reverse reaction of reductive carboxylation then consumes NADPH and with CO_2_ can transform 2OG to IC. This complete reaction of IDH2 is followed by citrate efflux from mitochondria, typically upon reductive carboxylation glutaminolysis (72, 73, 115, 116, 154, 195) (Fig. 1). However, an incomplete reaction, not requiring CO_2_, leads to a simple NADPH-driven reduction of 2OG to 2HG.

#### r-2HG formation by wild-type IDH1 and IDH2

There is no more controversy over whether the wild-type (wt) IDH1/2 enzyme is capable of such a reaction. We were among the first in demonstration that wt IDH2 produces 2HG (155). Also, the transfection of cells with wt IDH1 or wt IDH2 selectively increased r-2HG, despite its levels being 50- to 100-fold lower than those produced by overexpressed IDH1^R132H^-mutant enzymes (70). Indeed, the recombinant human IDH1 undoubtedly catalyzed the reduction of 2OG to r-2HG (70). Consequently, previous observations became explainable, such as when c-Myc-retransformed breast cancer tissues were found to contain substantial levels of 2HG (1–20 nmol/mg, *i.e*., up to ∼20 m*M*) in the absence of IDH1/2 mutations (168).

Glioblastoma SF188 cells also produce 2HG at hypoxia, again despite lacking the IDH1/2 mutations (183). Also, estrogen receptor-negative (ER−) breast carcinoma HTB-126/Hs 578T cells, and epithelial adenocarcinoma MDA-MB-231 cells, contain 2HG in the absence of IDH2 mutations and its formation substantially dropped upon IDH2 silencing (155). In hypoxia, s-2HG was induced (68), whereas r-2HG accumulation also occurred upon the depletion of its metabolizing enzyme, r-2HG-dehydrogenase (107).

It should be investigated whether each wt IDH1/2 molecule forms r-2HG with an ∼1000 lower turnover as “an error” during NADPH-dependent reductive carboxylation or whether there exists a specific pool of wt IDH1/2 proteins, distinct, for example, by having specific post-translational modifications, but still without mutations.

#### Mutant IDH1 and IDH2 as sources of r-2HG

**FIG. 2.**
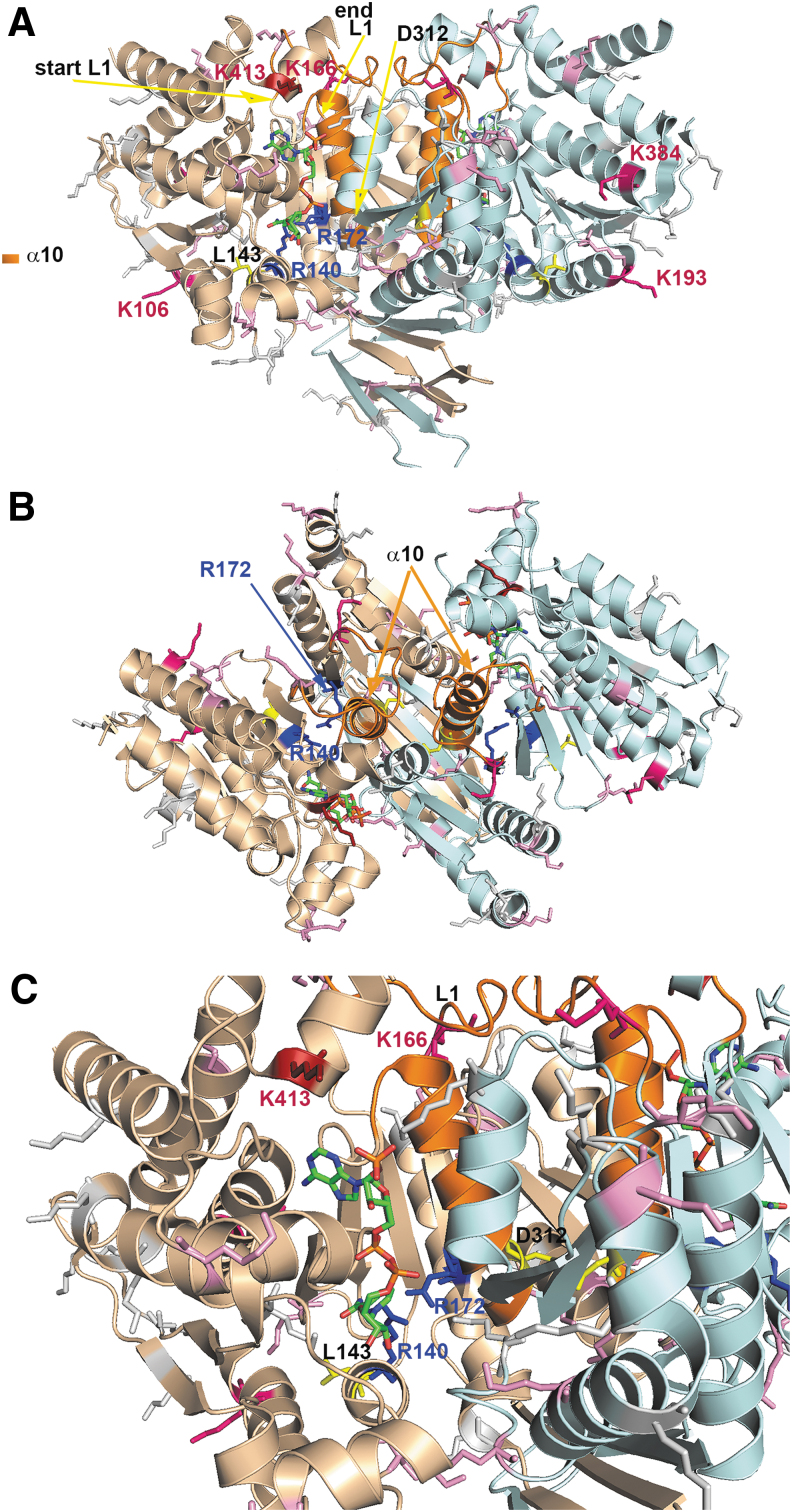
**Dimeric structure of IDH2. (A)** Side view, **(B)** top view, and **(C)** detail from **(A)** are shown for the structure of the human IDH2 dimer. The dimer is modeled with each monomer in a distinct color (*light brown*, *light blue*), while important features stabilizing the conformation of the reaction center (partly emphasized by a bound NADPH, *green* with *blue* and *red* atoms) are depicted, such as loop L1 of residues 152–167 and helix α10 (residues 311–326, *orange*). Also, lysines susceptible to inhibitory acetylation are highlighted in *shades of red* (*dark red* for the most proximal lysines to bound NADPH). An important D312 residue and L143 (aligning the opposite end of the NADPH binding site) are emphasized in *yellow*. The structure was derived from the published structure of the mitochondrial IDH2 mutant R172K, pdb code 5svn (184) and created using the PyMOL Molecular Graphics System Version 2.0 Schrodinger, LLC. Tentatively, the original Arg172 was put back (a mutagenesis function was applied with a most suitable rotamer of the arginine), being aware that mutant structure is slightly distinct from the wt IDH2 structure (see the Mutant IDH1 and IDH2 as Sources of r-2-HG section and the Specific Inhibitors of Mutant IDH1/2 section). wt, wild-type.

In human grade II/III gliomas (35, 36, 74, 76, 180), secondary glioblastomas (127), AML (16, 60, 103, 144), cholangiocarcinoma, chondrosarcoma (2), and in other cases of different tumor types (20, 54, 135, 192), heterozygous somatic missense mutations were found in IDH1 arginines of the catalytic site, such as (bold for most abundant) **R132H**/**C**/L/S and R100Q, or IDH2 arginines, such as **R140Q**/G/W/L and **R172K**/G/M/Q/T/S (20, 180) (Fig. 2). Mutant enzymes exhibit an impaired oxidative decarboxylation reaction (IC to 2OG) but render a partial reverse, that is, reductive reaction, exclusively forming the r-2HG enantiomer (54).

In wt enzymes, arginines form hydrogen bonds with both the α and β carboxyl of IC and thus ensure IC binding (158, 187). Substitutions of arginines decrease affinity for IC binding but increase it for NADPH (35, 36). With IDH1^R132H^, the resulting mutation prevents conformational changes between the initial IC binding and a pre-transition state (190). The IDH1^R132H^ enzyme is thus set to the so-called closed/active conformation (36), where H132 cannot interact with N271 of a “regulatory segment” (“segment α10”), that is, α-helix 271–286 (187), causing a 300-fold decrease in the catalytic efficiency relative to the wt IDH1 enzyme and a 1000-fold loss of affinity (38) for Mg^2+^.

Since r-2HG also preserves the transformed phenotype of cancer cells with IDH mutations (128), it should also have a regulatory role. Cells expressing mutant IDH1 accumulate less r-2HG relative to those expressing mutant IDH2 (181). Nevertheless, up to 1 to ∼30 m*M* r-2HG can be found (30, 36, 60). IDH1/2 mutants cause significant variations in the fluxes of 2OG, IC, and other metabolites, accompanied by redox changes to establish new NADPH/NADP^+^ equilibria in both mitochondrial and cytosolic compartments. Such compensations include increased glutaminolysis (121, 136). Cells are also sensitive to the inhibition of glutaminase (106, 145).

Three phenotypes were characterized: The first phenotype involved depleted 2OG but moderate r-2HG and was associated with the most common R132H and R132C IDH1 mutations; the second exhibited moderate 2OG levels and high r-2HG levels and was associated with IDH1^R132Q^; the third phenotype was characterized by depleted 2OG but again high r-2HG levels, being associated with R132L (108).

#### Specific inhibitors of mutant IDH1/2

The resulting changes induced by 2HG in chromatin and the cell differentiation state are mostly reversible (54, 99). Hence, in principle, they could be reverted by specific inhibitors for mutant IDH1/2. Indeed, specific inhibitors have been developed for mutant IDH1 (15, 38, 92, 122, 130, 131, 173, 184) or mutant IDH2 enzymes (179). Usually, they do not bind the mutated arginines except to an allosteric pocket of each monomer, which is not accessible in wt enzymes (35). Since in mutant IDH1 enzymes a regulatory segment α10 (187) is destabilized, and hence only partially ordered (184), there is an open accessible pocket space for the inhibitor. Moreover, bound Mg^2+^ protects the inhibitor binding to the wt enzyme. These properties determine which inhibitors are specific for the mutant IDH1 enzyme.

In contrast, IDH2 mutants are targeted by different drugs. This is because the IDH2 mutant is set to the closed/inactive conformation with stabilized α10. A specific IDH2 inhibitor was developed to bind to the IDH2 dimer interface (179). Surprisingly, tumors targeted by the specific IDH1 inhibitors have the ability to switch their mutagenesis toward unmutated IDH2, which is not affected, and *vice versa* (63, 69).

Nevertheless, AG-881 from Agios Pharmaceuticals was claimed to inhibit both mutant IDH1/2 in a common allosteric pocket (102). The inhibitor of mutant IDH1 ivosidenib (40, 130) and mutant IDH2 enasidenib (159, 160) exhibited positive responses in patients with relapsed or refractory gliomas, intrahepatic cholangiocarcinomas, and chondrosarcomas (48, 130) in phase I/II clinical trials. Ivosidenib was also tested in AML patients, but acquired resistance for these mutants was frequently developed (118). Enasidenib also induced remissions of AML (3, 159).

It seems that specific inhibitors should be designed for each mutation. For example, the mutant IDH1^R132Q^ was 10^5^-less sensitive to mutant inhibitors than IDH1^R132H^ (108). This was explained by the conformation of α-helices more closely resembling the wt enzyme.

### Other enzymes producing r-2HG

#### Hydroxyacid-oxoacid transhydrogenase/alcohol dehydrogenase iron-dependent isoform 1

Physiological mitochondrial metabolism involves both 2HG enantiomers (87). In mammalian mitochondria, hydroxyacid-oxoacid transhydrogenase (HOT), also known as alcohol dehydrogenase iron-dependent isoform 1 (ADHFE1; EC 1.1.99.24), forms r-2HG from 2OG, whereas it simultaneously converts 4-hydroxybutyrate to succinic semialdehyde (20). HOT/ADHFE1 thus competes with IDH2 for 2OG (162) (Fig. 1). ADHFE1 has been recognized as a breast cancer oncogene since it is upregulated by Myc *via* the enhancement of iron metabolism (113). Elevated ADHFE1 produced increasing levels of r-2HG, whereas ROS were also increased in conjunction with the elevated reductive carboxylation and NADPH consumption. r-2HG then concomitantly changed epigenetics as described below in the General Effects of 2HG section.

#### Glutathione-dependent glyoxylases

In mammalian cells, r-2HG is also produced from 5-aminolevulinate (24). The oxidative degradation of heme precursor 5-aminolevulinate converts it to 2HG through the action of the glutathione-dependent glyoxylase isoform 1 (GLO1) and isoform 2 (HAGH) (165). They have no known mutations or dysregulations in cancer.

#### Phosphoglycerate dehydrogenase

Also, human phosphoglycerate dehydrogenase (PHGDH; EC 1.1.1.95) has been reported to form r-2HG from 2OG (47). The PHGDH production of r-2HG increases at acidic pH. The expression of PHGDH is quite frequently enhanced in breast carcinomas (96, 132). This may provide a source of 2HG for breast cancer cells.

### Enzymes producing s-2HG

#### Lactate dehydrogenase

A noncanonical or side function of several enzymes also leads to the formation of s-2HG. Lactate dehydrogenase (LDH) is able to interconvert 2OG to s-2HG at the expense of NADH, specifically under hypoxia in normal or malignant cells (68, 70, 123) or acidic conditions (117). Note that under hypoxia, this stems from HIF transcriptome reprogramming. Nevertheless, the resulting s-2HG may provide a strengthening of certain HIF-evoked regulations and epigenetic changes by inhibiting 2OG-dependent dioxygenases.

At acidic pH, 2OG binds more stably to LDHA, with a concomitantly enhanced s-2HG formation (70). This contrasts with the r-2HG formation by a side reaction of IDH1/2, which is pH-independent. Thus, s-2HG is produced by LDH to reach approximately by two orders of magnitude less levels than those of r-2HG formed by mutant IDH1/2 (70). In contrast, the PHGDH production of r-2HG is pH dependent. When LDH forms s-2HG at the expense of NADH, glycolysis and concomitant oxidative phosphorylation (OXPHOS) respiration are slowed down due to the resulting NAD^+^ accumulation (70) (Fig. 1). Interestingly, a specific isoform C of LDH (LDHC) is a significant producer of s-2HG in the testis, where levels of up to 200 nmol/g s-2HG are found, compared with much lower levels in the liver (∼20 nmol/g) (41).

#### Malate dehydrogenase

s-2HG is generated during the conversion of oxaloacetate to l-malate by l-malate dehydrogenase, MDH2, a mitochondrial Krebs cycle enzyme (139), and also by the recombinant enzyme (70). A pH optimum was found at 6.6 for the formation of 2HG by MDH2, with the rate being almost half at pH 7.8 (117). Also, cytosolic MDH1 can form s-2HG as a side reaction. Both malate dehydrogenase (MDH) isoforms are considered the most frequent s-2HG sources.

### Degradation of 2HG and other reactions

#### Degradation of 2HG

The catabolism of 2HG diminishes its levels (45). The specific degradation of r-2HG to 2OG proceeds *via* r-2HG-dehydrogenase (EC 1.1.99.39) (94) localized to the mitochondrial matrix (1), whereas the s-enantiomer is catalyzed by the cytosolic plus mitochondrial enzyme of EC 1.1.99.2. A deficiency of r-2HG-dehydrogenase causes type-I glutaric academia (107, 129, 161). Since r-2HG-dehydrogenase accepts electrons from electron transfer flavoprotein:ubiquinone oxidoreductase (ETF:QOR) (161), deficiencies of this oxidoreductase also lead to a similar disease, glutaric acidemia type-II. The latter is frequently also caused by the mutant IDH2 R140G (86, 87).

The recombinant r-2HG-dehydrogenase has a turnover of 0.8 s^−1^ (44), whereas the mutant IDH1 has a turnover up to 1000 s^−1^ (36). If no other enzyme consumes r-2HG, its resulting accumulation should be enormous. The s-2HG-dehydrogenase reaction can be regarded as “correcting” side reactions of LDH and MDH (64).

#### Other reactions

Also, human glutamine synthetase ensures the amidation of r-2HG and s-2HG. The latter reaction leads to s-2-hydroxyglutaramate upon the parallel transamination of l-glutamine to 2-oxoglutaramate by LDH (64). Human ω-amidase can degrade s-2-hydroxyglutaramate back to s-2HG. Similarly, 2-oxo-succinamate, as a transamination product of l-asparagine, is converted by LDH to s-2-hydroxysuccinamate, which can be degraded by ω-amidase to l-malate. It is noteworthy that perturbing these pathways may also lead to tumor progression (64).

## Regulations and Signaling by 2HG

### 2HG and redox homeostasis in carcinogenesis

#### Redox homeostasis related to canonical IDH1 and IDH2 reactions

IDH1 supplies 2OG for cytoplasmic and nuclear dioxygenases that require 2OG as a co-substrate (66) and regenerates extramitochondrial NADPH, which is required for lipid biosynthesis and antioxidant protection. IDH1 also supplies NADPH for the constitutively expressed NADPH oxidase isoform 4 (NOX4), producing hydrogen peroxide (H_2_O_2_). Together with malic enzyme (ME) 1 and two enzymes of the pentose phosphate pathway (PPP; glucose-6-phosphate dehydrogenase [G6PDH] and 6-phosphogluconate dehydrogenase), IDH1 contributes to the cytosolic NADPH pool, being the main NADPH source in the brain and several other tissues (10).

**FIG. 3.**
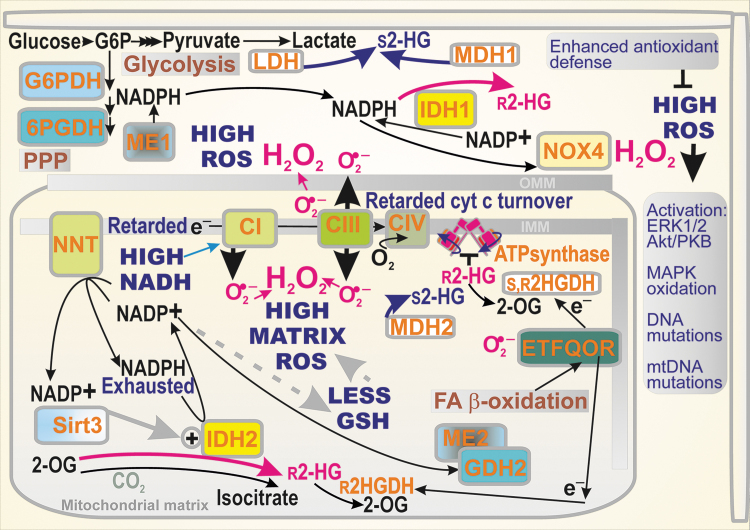
**Consequences of IDH1/2-related cancer metabolism for redox homeostasis.** A simplified scheme demonstrates the pro-oxidant character of reductive reactions of IDH1 and IDH2. Retarded electron (e^−^) transfer *via* the respiratory chain complexes CI, CIII, and retarded turnover of cytochrome *c*, together with feedback inhibition of proton pumping by the inhibited ATP-synthase with r-2HG (hypothetically also in humans) are the main inducers of elevated superoxide (O_2_^•−^) formation. In particular, a high NADH/NAD^+^ ratio leads to superoxide formation at the flavin I_F_ site of Complex I (not shown). Superoxide is dismuted by the matrix MnSOD/SOD2 and intermembrane space or cytosolic CuZnSOD/SOD1 (data not shown) into H_2_O_2_, the most prominent ROS. High ROS formation within the mitochondrial matrix depletes GSH, which requires NADPH for its synthesis. As a result, NADPH is instead depleted or rapidly diminished. The oxidated mitochondrial ROS equilibrium is spread toward the cytosol. Also, fast tumor growth contributes to depletion of cytosolic NADPH. Its pool is regenerated when cytosolic NAPDH is supplied by two PPP enzymes, G6PDH and 6PGDH but consumed again by the constitutively active NADPH oxidase NOX4 that produces H_2_O_2_ directly. Both r-2HG and s-2HG can stimulate not only the NRF2-reprogrammed elevated antioxidant defense that attenuates the cytosolic but also mitochondrial oxidative stress. Nevertheless, ROS being elevated even transiently can evoke the activation of redox-sensitive kinases, besides oxidizing DNA and mtDNA. 6PGDH, 6-phosphogluconate dehydrogenase; G6PDH, glucose-6-phosphate dehydrogenase; GSH, reduced glutathione; H_2_O_2_, hydrogen peroxide; mtDNA, mitochondrial DNA; NOX4, NADPH oxidase isoform 4; NRF2, nuclear factor erythroid 2-related factor; PPP, pentose phosphate pathway; ROS, reactive oxygen species; SOD, superoxide dismutase.

Also, the typical IDH2 mode is oxidative, that is, NADP^+^-dependent oxidative decarboxylation converting IC to NADPH and 2OG. This “forward Krebs cycle” direction is the typical reaction in nonmalignant cells. However, both enzymes are reversible, changing directions depending on the IC to 2OG and NADP^+^ to NADPH ratios, and presence of Mg^2+^ and CO_2_. This reversibility links these enzymes to redox homeostasis (Fig. 3). Thus, IDH2 may act in a “reverse” Krebs cycle mode in the reductive carboxylation reaction (72, 73, 115, 116, 154, 195) (Fig. 1). This reductive reaction of IDH2 as well as IDH1, including a side r-2HG formation, then shifts redox homeostases in mitochondria and cytosol toward the more oxidated state. These unavoidable conditions are due to the NADHP being instead exhausted in this reductive (reverse) mode since also the demand of NADPH for lipid synthesis is enormous in malignant cells.

In contrast, since NADPH is produced in the oxidative mode, such a “normal” IDH2 reaction thus substantially contributes to keeping the mitochondrial matrix in a reduced redox state and consequently prevents oxidative damage (75, 81, 82). IDH2 supplies the mitochondrial NADPH pool together with nicotine nucleotide translocase (NNT), ME2 (mitochondrial), and glutamate dehydrogenase (GDH) (Figs. 1 and 3). This pool serves for the regeneration of mitochondrial antioxidant systems, reduced glutathione (GSH) and reduced thioredoxin by glutathione reductase and thioredoxin reductase, respectively (72).

Consequently, IDH2 plays an important role in the ROS homeostasis (72) and in the prevention of apoptosis (61), such as that induced by heat shock (150) or in neuroprotection (87). Interestingly, a self-perpetuating antioxidant effect of IDH2 stems from the fact that deglutathinylation activates IDH2 at the prevailing reduced matrix glutathione level (81). In turn, the glutathionylation of IDH2 inhibits its activity when there is a substantial amount of oxidated glutathione in the mitochondrial matrix (81).

The regular Krebs cycle enzyme IDH3, structurally distinct from IDH2, then converts NAD^+^ irreversibly to NADH. The IDH3 reaction is allosterically positively regulated by Ca^2+^, ADP, and citrate, and negatively regulated by ATP, NADH, and NADPH (149). When the OXPHOS glutaminolysis takes place in cancer cells, the aconitase-IDH3 segment is frequently inactive (72, 195). This results in a decrease in the substrate pressure (NADH/NAD^+^) and mitochondrial superoxide formation.

#### 2HG affecting redox homeostasis

As discussed above, the predicted general effect of modes of r-2HG production is a shift toward the oxidated state. This is valid for both IDH1 and IDH2 and notably for their mutants, which exhibit much a higher turnover of r-2HG production. However, despite the decreased NADPH, GSH levels are maintained in the gliomas containing mutant IDH1/2 (46). This compensation includes the enhanced expression of key enzymes for glutathione synthesis, including cystathionine-β-synthase (CBS) (46).

Also, when aerobic glycolysis predominates and LDH or MDH1, 2 are allowed to provide a parasitic formation of s-2HG, the oxidated state predominates since the slowed down OXPHOS and slow mitochondrial respiration lead to the leakage of electrons to oxygen at specific sites of the respiratory chain and/or key dehydrogenases, thus forming an excessive amount of superoxide (14) (Fig. 3).

Only a disbalance leads to the so-called oxidative stress when ROS production significantly and permanently exceeds the antioxidant mechanisms (125). A general oxidative stress in a cell arises when the function of redox buffers and antioxidant enzymes is diminished, so that they no longer possess the ability to detoxify the produced ROS. A permanent character distinguishes this stress from repeatable redox signals. The direct pathological consequences are due to the oxidative stress, which reaches a certain threshold when there is an accumulation of oxidative products of biological constituents (oxidative modification of lipids by nonenzymatic lipid peroxidation or oxidative modification of proteins, such as carbonylation). This may initiate programmed cell death, such as apoptosis. Of course within a tumor, apoptosis would retard its growth. The tumor cells prevent this regress by overexpressing antioxidant systems.

A specific line of effects of oxidative stress is concerned with oxidative modifications of DNA and of more vulnerable mitochondrial DNA (mtDNA). Physiological mechanisms exist for DNA repair. However, in cancer (stem) cells, excessive DNA oxidation in synergy with insufficient DNA repair leads to the occurrence of somatic mutations, which are prerequisites for the origin of the primordial cancer cells. When the impairment of normal autophagy and notably autophagic mechanisms dealing with mitochondria also lead to the accumulation of products that were supposed to be cleared, this must have serious consequences for the cell. Again, this acts against carcinogenesis.

#### 2HG affecting redox signaling

ROS manifest dual functions as cancer promoters and cancer suppressors (124). The regulation of redox reactions impacts RAS-RAF-MEK1/2-ERK1/2 signaling related to carcinogenesis (154). Also, NADPH oxidases are ROS sources that promote or modulate this pathway. In contrast, redox signaling is involved in the p38 mitogen-activated protein kinase (MAPK) pathway that suppresses cancer by oncogene-induced senescence, inflammation-induced senescence, replicative senescence, contact inhibition, and DNA-damage responses (154). Nevertheless, MAPK also plays a procarcinogenic role (55). Another branch of redox signaling initiated with electrophiles is provided by the KEAP1-nuclear factor erythroid 2-related factor (NRF2) transcriptome upregulation of antioxidant and other genes (138).

Elevated ROS are able to control the transition from proliferating to quiescent phenotypes and to signal the end of proliferation. Suppression of these higher ROS levels in tumor cells should allow sustained proliferation. The upstream elements responsible for H_2_O_2_-induced extracellular-related kinase (ERK) 1/2 and protein kinase B (Akt) activation remain poorly characterized, but a potential role has been postulated for receptor and nonreceptor protein tyrosine kinases as triggers that initiate such events (124). The pathway involving PI3K and Akt is also redox-regulated through the oxidation of cysteine residues in phosphatases (*e.g*., phosphatase and tensin homolog [PTEN] and protein phosphatases 1 and 2). Akt then regulates an array of downstream targets including pro- and antiapoptotic members of the BCL2 family, caspase-9, forkhead box protein O (FOXO) family members, GSK-3β, and mammalian target of rapamycin (mTOR) (32).

It was also suggested that the cancer cell phenotype persists because of selective MAPK oxidation in mitochondria (55). Thus, H_2_O_2_ reportedly induces MAPK transfer to mitochondria, where it co-localizes with upstream kinases (MAPKKs). Subsequent oxidation of conserved cysteines in MAPK results in MAPK-MAPKK translocation to nuclei with consequent ERK1/2 and p38-JNK1/2 activation and a concomitant increase in ERK1/2-mediated cell proliferation and p38-JNK1/2-mediated cell cycle arrest (55). It has been hypothesized that because “dysfunctional” mitochondria in cancer cells may not generate excess ROS, the above-mentioned MAPK oxidation is disrupted and cells remain in proliferation mode.

Lower mitochondrial respiration is triggered by metabolic constraints and, along with the accumulation of mutations in mtDNA in some tumors, is associated with high-level ROS generation in mitochondria (71). This promotes genetic instability in tumors and favors growth, chemotherapeutic escape, and tumor stage progression.

As mentioned above, a larger extent of NADPH depletion results in a disbalance of redox equilibria toward oxidative stress. Of course, its actual occurrence depends on simultaneous changes in antioxidant defense, which can even be improved, for example, by the activation of NRF2-mediated expression of the antioxidant proteins. An increased flux *via* PPP was also found to support r-2HG formation by mutant IDH1 since the two PPP enzymes produce NADPH, the 1^st^ PPP enzyme, G6PDH, and 6-phosphogluconate dehydrogenase (58). Undoubtedly, the availability of NADPH controls the extent of r-2HG formation. A constitutive NOX4 then produces more H_2_O_2_ upon the increased PPP flux and elevated NADPH synthesis.

#### Specific redox homeostasis in hypoxia

In hypoxic cells, the ratio of NADH/NAD^+^ (substrate pressure) increases (57). This leads to enhanced superoxide formation at the flavin I_F_ site of Complex I and perhaps also by mitochondrial dehydrogenases (14). Moreover, as a direct consequence of HIF transcriptome reprogramming promoting aerobic glycolysis (the Warburg phenotype), the suppressed OXPHOS is usually linked to a slow Krebs cycle turnover, but an increased accumulation of 2OG (183). Since the acidification also increases due to the enhanced lactate formation and carbonic anhydrase reaction in hypoxia, conditions are set for the described parasitic reactions of LDH and MDH, forming s-2HG. Since there is an interference of 2HG with HIF, the resulting complex situations are described in the Interference with HIF Signaling section.

#### The role of mitochondrial sirtuins in regulation of IDH2

Lysine acylation is a common reversible post-translational modification associated with regulatory mechanisms of enzymes and proteins in general. Most frequently, acetylation, malonylation, succinylation, glutarylation, and so on, leads to the inhibition of protein function since they eliminate the positive charge of lysine (152). Mitochondrial lysine deacetylation is controlled by the NAD^+^-dependent deacetylase sirtuin 3 (SIRT3) (39, 142, 174). SIRT3 activity promotes OXPHOS and catabolic metabolic pathways and, due to its NAD^+^ dependence, is controlled by the redox state.

A higher substrate pressure deactivates SIRT3, whereas SIRT3 should be activated during the operation of redox shuttles and/or OXPHOS glutaminolysis, when the substrate pressure is lower (NAD^+^ higher). The ablation of SIRT3 causes pleiotropic effects in cancer but typically SIRT3 acts as a tumor suppressor protein (82, 193). Loss of SIRT3 leads to increase in proliferation and tumor growth, resulting from the concomitantly increased mitochondrial superoxide formation (164).

Acetylated IDH2 exhibits a reduced activity and, in turn, SIRT3-mediated deacetylation elevates the forward NADP^+^-dependent IDH2 reaction (194). The deacetylation of IDH2 prevents the oxidated state of the mitochondrial matrix *milieu* and helps to maintain the mitochondrial glutathione levels. Moreover, IDH2 acetylation was associated with a disturbance of the homodimeric IDH2 structure (200). Thus, the IDH2 K413Q mutant, simulating acetylation in the sense of the positive charge vanishing, also exhibited a reduced dimerization (200). There is also disagreement over the acetylation itself (156). Suggestions include the acetylation resulting from a nonenzymatic (uncatalyzed) reaction of acetyl-CoA at alkaline pH, which typically occurs in the matrix of respiring mitochondria (177).

SIRT3 was suggested as a target in breast cancer since higher SIRT3 expression was correlated with a poorer prognosis for patients with grade III breast carcinoma (171). Besides the reported interference with redox homeostasis (169) and mitochondrial biogenesis (170), this phenomenon may also stem from SIRT3-mediated activation of 2HG production by IDH2 (156). Also, another mitochondrial sirtuin, SIRT5, was found to ensure the desuccinylation of IDH2 to activate the enzyme (199). Consistent with the antioxidant role of IDH2, the ablation of SIRT5 led to increasing cellular ROS.

#### The role of sirtuin 1 in regulation related to 2HG

Among all sirtuins 1–7 (isoforms 3,4,5 being mitochondrial), the NAD^+^-dependent deacetylase sirtuin 1 (SIRT1) deacetylates numerous proteins mainly in the nucleus and also in the cytosol. Consequently, SIRT1 is involved in numerous cellular regulations (*e.g*., transcription factors, p53, FoxO proteins, PPARγ, PGC1α, and nuclear factor kappa-light-chain-enhancer of activated B cells [NF-κB]), including histones. SIRT1 generates nicotinamide, while the acetyl group of the protein substrate is transferred to cleaved NAD, generating O-acetyl-ADP ribose (137). In several types of cancer, SIRT1 is elevated and may serve as a tumor promoter. In contrast, in certain situations, SIRT1 may act as tumor suppressor (137).

#### Possible signaling mediated by r-2HG

The development of malignancy is inevitably related to metabolic reprogramming. Studies of cancer-specific metabolism have demonstrated that besides the shifts in metabolic pathways, certain metabolites play an information signaling role. Notably, Krebs cycle substrates and derived metabolites such as succinate, fumarate, itaconate, acetyl-CoA, and both enantiomers of 2HG exhibit such a nonmetabolic signaling function (138). The latter refers to the inducer-mediated event resulting in an altered expression of specific sets of genes or changes in the epigenome. This aspect will be described in the next sections for 2HG. The finding (21) that 2HG activated the mTOR pathway is one such signaling role. However, there are sure to be other information signaling pathways affected by 2HG enantiomers.

### Metabolic regulations

The most important effect of 2HG lies in the ability to inhibit the chromatin-modifying enzymes (see the Antagonism of 2HG in epigenetic changes section). This effect dysregulates the cell's gene expression, which otherwise supports differentiation in normal nonmalignant cells. As a result, 2HG promotes carcinogenesis by stabilizing malignant cells in an undifferentiated state similar to stem cells (51, 100, 144, 173, 186). Numerous other effects of 2HG stem from the dysregulated metabolism, which we will discuss first.

#### General effects of 2HG

There is a very wide range of cancer cells. One can expect (140) that 2HG might also activate the NRF2, downregulate p53 (73), inactivate pyruvate dehydrogenase (PDH) enzymes (143), and decrease the demethylation of DNA and histones, causing the so-called hypermethylation (20, 29, 34, 35, 37, 51, 54, 88, 98, 99, 100, 135, 144, 164, 173, 186). The resulting metabolic reshuffling typically involves increased glutaminolysis (46) and may also produce an increase in glycolysis (HIF activation even under aerobic conditions) and an increase in fatty acid β-oxidation (140). As a rule, this is accompanied by a typically increased ROS production and dysregulation of redox homeostases and redox signaling. Moreover, in *Caenorhabditis elegans*, 2HG was found to inhibit ATP-synthase, a phenomenon that can also occur in glioma cells with mutant IDH1/2 (52).

#### Synergy of 2HG-producing enzymes with other enzymes

A synergy exists for other enzymes with processes producing 2HG. A typical example is mitochondrial glutaminase, a key enzyme of glutaminolysis. For example, AML cells are dependent on glutamine and exhibit an increased expression of glutaminase, specifically its isoform GLS1 (glutaminase 1) (106). When glutaminase was inhibited, glutamate levels decreased and the growth of AML cells was inhibited. Also, 2HG concentrations declined specifically in AML cells with IDH1/2 mutants (106). Importantly, the inhibition of glutaminase mostly suppresses tumorigenesis, at least partially.

Branched-chain amino acids (BCAA) are metabolized by BCAA aminotransferases (cytosolic BCAT1 and mitochondrial BCAT2), transferring their α-amino group to 2OG. A key role of BCAT1 in AML was determined by experiments with BCAT1 ablation, which increased 2OG and this in turn increased PHD2-mediated HIF-1α degradation (134). As a result, leukemic cells lost the ability to survive and stopped their growth. In contrast, BCAT1 overexpression caused DNA hypermethylation *via* ten-eleven translocation methylcytosine dioxygenase (TET) due to the decreased 2OG (see the Antagonism of 2HG in epigenetic changes section).

Lipoyl transferase 1 (LIPT1) is another enzyme whose deficiency elevates 2HG levels. LIPT1 is essential for the lipoylation of PDH subunit E1, which forms acetyl CoA from pyruvate and thiaminepyrophosphate. Therefore, LIPT1 tunes the balance between the oxidative and reductive glutaminolysis (115, 116), promoting the oxidative mode at a higher activity of LIPT1 (119). The ablation of LIPT1, such as in patients with lactic acidosis, causes a blockage of pyruvate oxidation by PDH, consequently increasing the pyruvate conversion to lactate and transamination of alanine with pyruvate by the aminotransferase reaction. Since 2OG dehydrogenases should also be lipoylated for a proper function, 2OG-dehydrogenase (2OGDH) is also blocked. This results in increases in glutamate and proline.

Altogether, the ablation of LIPT1 leads to the elevation of both 2HG enantiomers, due to the ongoing shift toward reductive carboxylation glutaminolysis. In this reaction, its first step can synthesize r-2HG in the absence of IDH2 mutations. Thus, in patients with lactic acidosis, r-2HG (300 ng/mL *vs*. 200 ng/mL in healthy subjects) and to a greater degree s-2HG (400 ng/mL *vs*. 100 ng/mL in healthy subjects) were elevated in plasma as well as in fibroblasts (119).

When 2OGDH and/or lipoic acid synthase (LIAS) were ablated, both r-2HG and s-2HG were elevated, also due to the 2OG accumulation (19). The latter could also be simulated by supplying dimethyl-2OG to cells (151). Under these conditions, s-2HG was formed by LDH, since its inhibitor oxamate inhibited the formation of s-2HG, and also by MDH1, 2. Only s-2HG inhibited PHD2, thus stabilizing HIF-1α independent of hypoxia (19, 31).

### Antagonism of 2HG in epigenetic changes

#### Inhibition of 2OG-dependent dioxygenases

**FIG. 4.**
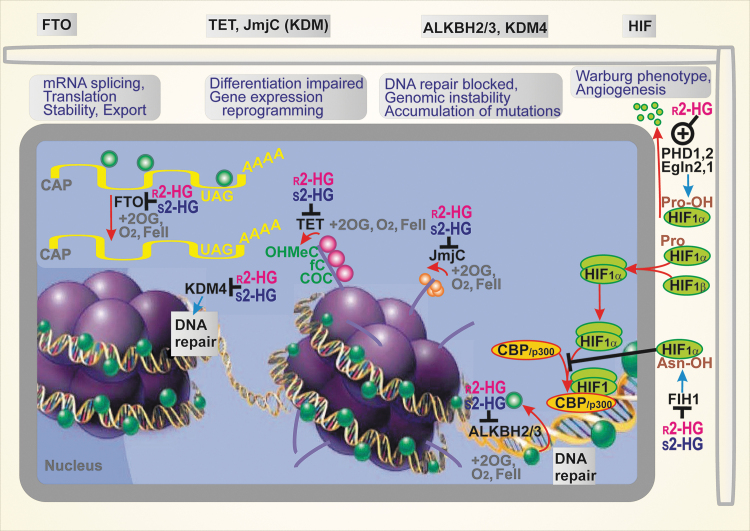
**Consequences of 2HG-mediated inhibition of 2OG-dependent dioxygenases and prevention of HIF transcriptome reprogramming *via* PHD/EglN activation by 2HG.** Epigenetic changes, specifically the hypermethylation of histones, leading to impaired differentiation and malfunctioned gene expression induced by both 2HG enantiomers, are stimulated as a result of the 2HG inhibition of the TET DNA demethylases and Jumonji histone lysine demethylases (JmjC/KDM). Likewise, the hypermethylation of DNA that blocks DNA repair occurs due to the 2HG inhibition of ALKBH2,3 enzymes (DNA repair enzymes of the AlkB family) and KDM4. Moreover, mRNA splicing, translation, and mRNA stability can be affected by 2HG's inhibition of the fat mass and obesity-associated protein (FTO), which otherwise catalyzes the demethylation of N6-methyladenosine when unblocked. In contrast, the prolyl-hydroxylase domain (PHD/EglN) enzymes, which degrade HIF-1α in the presence of oxygen, are stimulated by 2HG enantiomers, which mimic 2OG as a cofactor. Nevertheless, the other HIF system regulator, the factor-inhibiting HIF (FIH), is blocked by 2HG. *Red, orange spheres—*methyls of histones; *green spheres—*methyls of DNA. COC, carboxylcytosine; fC, formylcytosine; HIF, hypoxia-inducible factor; JmjC, Jumonji histone lysine demethylases; mRNA, messenger RNA; OHMeC, 5-hydroxymethylcytosine; PHD/EglN, proline hydroxylase domain enzyme (EglN); TET, ten-eleven translocation methylcytosine dioxygenase.

**FIG. 5.**
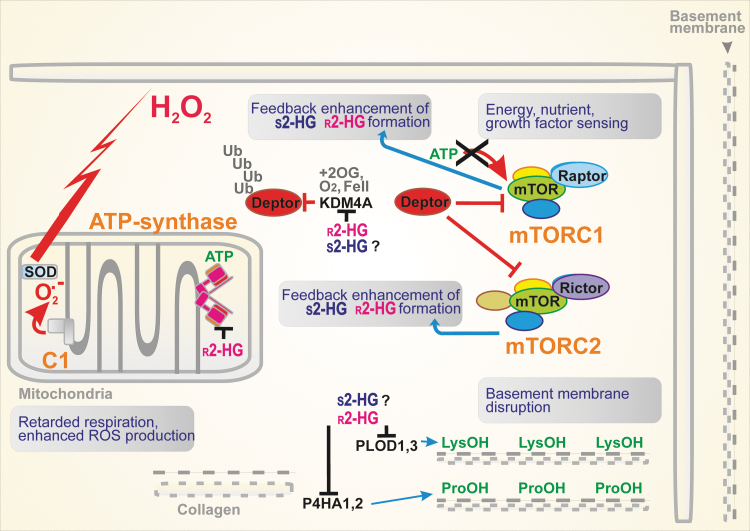
**2HG interference with the mTOR pathway and collagen maturation.** Since the 2OG-dependent dioxygenase KDM4A stabilizes DEPTOR, the endogenous negative regulator of mTOR, and since the 2HG-mediated inhibition of KDM4A releases DEPTOR by preventing its polyubiquitylation, the activation of both mTORC1 and mTORC2 is prevented. Consequently, the mTOR pathway is blocked. Otherwise, the activation of the mTOR pathway promotes cell proliferation and survival and consequently carcinogenesis. Since r-2HG inhibits KDM4A, DEPTOR is degraded and mTORC1/2 is activated, this inhibition causes an enhancement of carcinogenesis. Alternatively, since r-2HG was found to inhibit the ATP-synthase in *Caenorhabditis elegans*, supposing that this also proceeds with human ATP-synthase, one can also predict the activation of mTOR by the decreased ATP. This inhibition also contributes to higher mitochondrial superoxide formation. Since other 2OG-dependent enzymes participate in collagen maturation, this is also affected by r-2HG. Thus, procollagen-lysine 2-oxoglutarate 5-dioxygenase PLOD1 and PLOD3 and prolyl 4-hydroxylase P4HA1 and PHA3 stabilize the triple helix of collagen *via* the respective hydroxylations. Consequently, the inhibitory r-2HG blocks the maturation of collagen and disrupts the stability of the basement membrane, thus promoting tumor growth and metastases invasion. DEPTOR, DEP domain-containing mTOR-interacting protein; mTOR, mammalian target of rapamycin.

A small molecule, such as 2HG, influences enzymes requiring 2OG as a substrate or co-factor. Since a class of such enzymes regulates epigenetics, the 2HG dysregulation of epigenetics is the ultimate mechanism of accelerated cancerogenesis (20, 29, 34, 35, 37, 54, 98, 164), besides activation of mTOR pathway (Figs. 4 and 5). There are >70 2OG-dependent dioxygenases that promote histone demethylation when functional. Theoretically, all of them may be targeted by both inhibitory 2HG enantiomers, hence 2HG should increase histone methylation up to a hypermethylated state (88, 99, 186).

In particular, the myeloid tumor suppressor TET DNA demethylases, such as TET1, TET2, and TET3, provide 5-methyl-cytosine hydroxylation followed by the formation of 5-formyl-cytosine and 5-carbonylcytosine (186). The resulting base pair mismatches with guanine are subjected to base excision repair, leading to the demethylation of DNA. For example, TET2 is potently inhibited by r-2HG (85). Interestingly, s-2HG is a more potent inhibitor of 2OG-dependent dioxygenases than r-2HG (85, 99, 186). s-2HG may be their natural physiological regulator.

The other enzyme family targeted by r-2HG includes the Jumonji histone lysine demethylases (JmjC), a structurally diverse family of more than 30 2-OG dependent dioxygenases. JmjC remove methyl groups from the N-side chain of lysine residues in histones. The reaction sequence first involves the hydroxylation of the N″-methyl group, forming an unstable hemiaminal intermediate, and in the second step, it splits into the demethylated lysine and formaldehyde (104). The KDM4A and KDM4B JmjC enzymes were also implicate in DNA repair (162).

Elevations in DNA and histone methylation induced by 2HG have other important consequences, such as defects in DNA repair by homologous recombination (162). Both 2HG enantiomers inhibit DNA repair enzymes of the AlkB family, ALKBH2 and ALKBH3 (25). These defects sensitize the inhibition of poly(ADP-ribose) polymerase (PARP). Hence, the use of PARP inhibitors was suggested as another therapeutic strategy for cancers with mutant IDH1/2 (114, 162).

Interestingly, the effects of r-2HG in astrocytes with mutant IDH led to the indirect reactivation of telomerase reverse transcriptase (TERT), and as a result, transformation and immortalization were supported (120). r-2HG caused an increased methylation of histone lysines and promoted c-Myc/Max, both at the promoter of the *Tert* gene.

Also, hematopoietic stem cells maintain their stem cell character by maintaining a glycolytic (Warburg) phenotype, but their differentiation requires OXPHOS, as proven by the lack of differentiation upon ablation of the Rieske iron–sulfur protein of mitochondrial Complex III of the respiratory chain (5). Such ablation led to an r-2HG increase together with a hypermethylation of DNA and histones. Specific attention should be paid to the increased methylation in CpG islands, where for example, tumor-suppressive microRNAs (miRNAs), such as 148A, are encoded. Hypermethylation causes transcription silencing and hence reverses the tumor-suppressive role of miRNA 148A (93).

#### Inhibition of necroptosis

Necroptosis is a type of cell death that may be programmed to exhibit a necrotic phenotype (27). The typical mechanism involves induction by tumor necrosis factor-α (TNFα) upon its binding to the TNFα receptor complex. Such a death signal activates the receptor-interacting protein 1 (RIP1) and recruits RIP3, alongside the formation of the so-called necrosome. Subsequently, RIP3 is autophosphorylated and binds the mixed lineage kinase domain-like (MLKL) protein. Phosphorylated MLKL diffuses to the plasma membrane and initiates necroptosis (27). It was demonstrated that 2HG stimulates hypermethylation of the RIP3 promoter (191). It is amplified due to the ability of 2HG to bind to DNA methyltransferase 1 (DNMT1). Consequently, 2HG can inhibit the necroptosis since the levels of RIP3 protein are reduced. As a result, this mechanism belongs to those promoting tumorigenesis.

As for apoptosis, glioma cells containing mutant IDH1 exhibit more apoptosis upon the inhibition of Bcl-xL, thus being more vulnerable to this inhibition (79). Both wt IDH1/2 in the oxidative mode have a profound antioxidant and hence antiapoptotic role. A higher extent of the reductive mode of their reaction then leads to a lower apoptosis protection. This was simulated, for example, by silencing IDH2 in HeLa cells (150). Also, the sensitivity of HeLa cells toward apoptosis induced by ionic radiation (90) as well as TNFα and anticancer drugs was markedly elevated upon silencing IDH2 (80).

#### Interference with the mTOR pathway

The mTOR is a serine/threonine kinase, forming complexes with Raptor or Rictor, that is, mTORC1 and mTORC2, respectively. These complexes are regulated by amino acid and energy (ATP) levels. This enables mTORC1 to regulate cell growth and/or autophagy and mTORC2 to determine cell survival (21) (Fig. 5). In cancer cells, both mTORC1/2 are frequently activated by upstream negative modulators disabled by mutations. The modulators of the mTORC1/2 complex, tuberous sclerosis complex TSC1–TSC2 heterodimers, are inhibited by the PI3K/AKT signaling pathway. The TSC2 contains the GTPase-activating protein domain, whereas TSC1 stabilizes the heterodimer. As a result, the TSC1–TSC2 complex downregulates a small G-protein Rheb. Since Rheb is an activator of mTORC1, the mTORC1 activity is inhibited (21).

Since mTORC1/2 activation promotes cancerogenesis, so does the identified 2HG inhibition of KDM4A, a 2OG-dependent dioxygenase of the Jumonji family of lysine demethylases (21). Since KDM4A associates with one of the negative modulators, the DEP domain-containing mTOR-interacting protein (DEPTOR), the 2HG-mediated inhibition of KDM4A releases DEPTOR and activates mTORX1/2. Interference with the mTOR pathway was also reported for *C. elegans* (52).

#### Disruption of the cytoskeleton architecture

Procollagen-lysine 2-oxoglutarate 5-dioxygenase PLOD1 and PLOD3 and prolyl 4-hydroxylase P4HA1 and PHA3 stabilize the triple helix of collagen *via* the respective hydroxylations. Since r-2HG inhibits these enzymes, consequently, it blocks the maturation of collagen and disrupts the stability of the basement membrane (54). These events promote tumor growth and invasion of metastases (Fig. 5).

#### Interference with HIF signaling

The initiation of HIF reprogramming of the transcriptome involves the inhibition of prolyl hydroxylase domain enzymes (PHD1/EglN2, PHD2/EglN1, and PHD3/EglN3) by the decreasing oxygen in hypoxia. There is disagreement over whether HIF is stabilized or degraded by 2HG enantiomers (6). Originally, r-2HG was reported to potentiate PHD functions in astrocytes, so it should prevent the HIF responses (85) (Fig. 4). As a result, astrocytes proliferation was enhanced. The potentiation of PHD function by r-2HG was also observed *in vitro* (31, 167). Nevertheless, constitutively active HIF was observed in glioblastomas with mutant IDH1, and it has been hypothesized that the activity of PHD2 is inhibited by 2HG (198). However, the reported phenomena might involve an indirect inhibition of PHD2. For example, the ascorbate depletion may inhibit PHD2 (143). Another explanation may be based on the recent finding that the tH3K27 histone demethylase KDM6A/UTX directly senses oxygen and its loss thus prevents demethylation and blocks cell differentiation (23).

*In vitro* s-2HG inhibited PHD2 at ∼400 μ*M* (31), which may be too high to reach *in vivo*. This s-2HG accumulation did not affect the HIF responses (68). Nevertheless, independent of hypoxia, the accumulation of s-2HG was found to be associated with HIF activation (19). This was observed with ablated 2OGDH, which promoted s-2HG formation by LDH and MDH2. Since enzymes for phosphocholine synthesis such as choline kinase are upregulated when HIF is activated, 2HG inhibits the synthesis of phosphocholine and phosphoethanol amine (175). As a result, phospholipid metabolism is also altered in gliomas bearing IDH1/2 mutations.

## 2HG as an Oncometabolite

### Promotion of carcinogenesis by 2HG

#### Gliomas

IDH1/2 mutations should arise during embryonic development due to the somatic mosaic of mutant IDH1/2-expressing cells, such as IDH1 R132H/C/L/S or R100Q and IDH2 R140Q/G/W/L or R172K/G/M/Q/T/S, which are common mutations in gliomas (bold are the most frequent) (20, 180). This is accompanied by loss-of-function mutations of the p53 protein (110). A specific human isoform of glutamate dehydrogenase 2 (GDH2) was also reported to promote glioma. Since unlike GDH1, GDH2 is not inhibited by GTP, this enables the otherwise deficient 2OG input into the Krebs cycle to be replaced by converting glutamate to 2OG (178). Glutamate can be made from 5-oxoproline, resulting from a cleavage of dipeptide metabolites such as γ-glutamyl amino acids. An increased uptake of the latter was found in IDH1^R232H^ p53^−/−^ cells after the overexpression of GDH2, but not GDH1 (178).

Typically, millimolar concentrations of 2HG are found in gliomas bearing IDH1/2 mutants (Table 1). *In vivo* magnetic resonance (MR) imaging using echo-planar spectroscopic imaging dual-readout alternative gradients (DRAG-EPSI) detected 5 m*M* 2HG before surgery and 3–6 m*M* after surgery (4). Similar concentrations were found using long echo time MR spectroscopy with semi-localization by adiabatic selective refocusing. It was recognized that gliomas bearing IDH2 mutants accumulated more 2HG than those with IDH1 mutants (9, 148).

**Table 1.** Estimated Concentrations of 2-Hydroxyglutarate in Tissues or Cells and Body Fluids

Cancer type	Tissue 2HG concentration (μM)	Serum, urine, CSF 2HG concentration (μM)
Glioma		
Mutant IDH1,2	1000–30,000 (30, 36, 60)	CSF: 14.5–25.5 (7)
	800–11,000 (97) MALDI-TOF	Serum: 0.2–1.9 (49)
	350–9000 (77) LC/ESI/MS/MS	Urine: 0.007–0.1 (49)
Mutant IDH1	5000 (4) DRAG-EPSI NMR	
	1700–2600 (126) ^1^H&^13^C NMR 900 MHz	
	11,000 (130)	
wt tissue	200–400 (97) MALDI-TOF	Serum: 0.2–1.87 (49)
AML		
Mutant IDH1,2		Serum: 3.5–7.0 (16)
	Cells:	4.3–5.4 (159)
IDH1^R132H^	2600–14,300 (60)	
IDH1 R132C	12,200–23,300 (60)	
Mutant IDH2	26,800–32,500 (60)	
wt	20–700 (60)	6.7 (16)
Mutant IDH1,2		Urine: 20–80 (16)
Myeloma	1000–4000 (59) cells	Bone marrow supernatant 0.25–4.0 (59)
Breast cancer		
	500–20,000 (168)	Serum: 13 (50)
		CSF: 15 (7)
Stage IV		0.15 (50)
Healthy		0.011 (50)
Healthy		Serum: 0.7 (50)
Colorectal	150 (62)	
	100–700 (33)	
Renal cell carcinoma	3000 (147)	
Lactic acidosis		
r-2HG		Serum: 2.0 (119)
s-2HG		2.5 (119)
Healthy r-2HG		1.3 (119)
Healthy s-2HG		0.8 (119)

Reported amounts of 2HG were converted to concentrations on the assumption of 1 g being 1 mL and based on 200 μm^3^ volume of lymphocyte (AML cells).

2HG, 2-hydroxyglutarate; AML, acute myeloid leukemia; CSF, cerebrospinal fluid; DRAG-EPSI, echo-planar spectroscopic imaging dual-readout alternative gradients; IDH, isocitrate dehydrogenase; MALDI-TOF, matrix assisted laser desorption/ionization - time-of-flight; r-2HG, r-2-hydroxyglutarate; s-2HG, s-2-hydroxyglutarate; wt, wild type.

Matrix assisted laser desorption/ionization - time-of-flight analyses detected r-2HG within the 0.8–11 m*M* range in 4-μm frozen slices of brain tumors containing IDH1/2 mutations, whereas 0.2–0.4 m*M* r-2HG was detected in wt tumor tissue slices (97). This is comparable to concentrations of 0.35–9 nmol/mg tissue found by using liquid chromatography electrospray ionization tandem mass spectrometry in an IDH1/2 mutant bearing gliomas *versus* 0.5 pmol/mg up to 0.12 nmol/mg of wt gliomas (77). Concentrations of 1.7–2.6 m*M* 2HG were estimated, using combined 900 MHz ^1^H- and ^13^C-NMR analyses of extracts from IDH1-mutated brain tumor tissues (126).

Changes in the expression of other genes affect patient's prognoses and survival since they may induce positive or negative effects. Typically, tumor suppressor genes exhibit an increased expression in gliomas with mutant IDH1, whereas the expression of oncogenes declines (67). For example, gene expression of insulin-like growth factor-binding protein 2 (IGFBP) is downregulated by DNA methylation promoted by 2HG formed by mutant IDH1 (67). Moreover, prognoses are worse for patients with gliomas with a low expression of insulin-like growth factor binding protein 2 (67).

#### Acute myeloid leukemia

Ivosidenib (commercially Ibsovo) was reported to have a 40% response in AML patients. Nevertheless, since IDH2^R140Q^ is the most frequent mutation found in AML and IDH2 mutations were also found in angioimmunoblastic T cell lymphoma (20, 197), enasidenib was developed as inhibitor of mutant IDH2, inducing molecular remissions (159). It was suggested for AML patients that levels of 2HG in serum exceeding 1 μg/mL might indicate the presence of IDH1/2 mutations. Among a cohort of 200 such patients, about 25% indeed exhibited these IDH1/2 mutations, while a threshold of 0.5 μg/mL was identified for 2HG (16). Elevated 2HG levels were also found in urine, bone marrow aspirates, and aspirate cell pellets. Moreover, the progression of standard chemotherapy was associated with decreasing serum levels of 2HG, supporting a prognostic potential of 2HG (16). The ability of mutant-IDH1 inhibitors to provide effects in AML having mutant-IDH2 stems from the ability to switch their mutagenesis toward unmutated IDH1, which turns to be the right target (63, 69).

Also, the progression of asymptomatic precursor plasma cell malignancies to symptomatic multiple myeloma was associated with elevated 2HG (59). Another lymphoma stems mostly from R172 mutations of IDH2, angioimmunoblastic T cell lymphoma, a subtype of nodal peripheral T cell lymphomas (91).

#### Breast cancer

Like other types of cancer, breast cancer also undergoes metabolic reprogramming (17, 37, 164, 166, 168) and possesses a modified chromatin and tumor microenvironment in which the antitumor immunity can be suppressed. PHGDH has also been identified as a breast cancer oncogene (96, 132). PHGDH and possibly other sources, such as ADHFE1, besides the nonmutant IDH2 (155) and IDH1 are responsible for elevated 2HG levels in breast carcinoma in the absence of the IDH1/2 mutant enzymes (166, 168). The enhanced 2HG levels correlated with Myc signaling (168). Also, the ablation of ADHFE1 decreased 2HG (168).

It was recently demonstrated that Myc-induced ADHFE1 forming r-2HG is the main cause of the resulting metabolic reprogramming involving reductive carboxylation glutaminolysis and enhancing mesenchymal transition upon changed epigenetics of breast cancer cells. Similar changes, such as those that occur in gliomas, have been observed (112, 113). These changes are consistent with findings of substantial levels of 2HG in the body fluids of breast cancer patients. Thus, ∼15 μ*M* r-2HG was found in the cerebrospinal fluid (CSF) of breast cancer patients after surgery, similar to patients with lung cancer (7), which may even be comparable to some patients with glioma bearing IDH mutations (14.5–25.5 μ*M* r-2HG in CSF) (7). A rare breast cancer subtype, solid papillary carcinoma with reverse polarity, has been also found to develop due to mutant IDH2 (28).

#### Other cancer types

IDH mutations were identified in ∼20% of cholangiocarcinomas (12). In a few cases, other types of cancers (78) carried IDH1/2 mutations, such as paraganglioma (53), colon cancer (153), prostate cancer, and lung cancer (146). Chondrosarcomas contain abundant 2HG (2). The kinetics of 2HG formation could be assessed by a hyperpolarized MR imaging technique (141).

In colorectal cancer cells, the epithelial–mesenchymal transition is induced by r-2HG (33). Moreover, it was found that the progression of colitis to colon cancer is associated with increased r-2HG in urine, and it was concluded that urine r-2HG is a good potential biomarker (62). Since r-2HG-dehydrogenase is also upregulated by HIF-1α, a reduced transcription of r-2HG-dehydrogenase at inhibited HIF responses contributes to the progression of colon cancer (62).

IDH mutations may also contribute to prostate cancer since a benign prostatic epithelial is transformed into a malignant one by certain miRNAs, which in turn are promoted by IDH1^R132H^ mutations (196). Also, up to 5% of patient samples of melanoma contained IDH1^R132C^ or IDH1^R132S^, which co-existed in 3% of samples with NRAS mutations (95).

Elevated IDH1 expression, including the common R132H mutations, was found in non-small-cell lung cancer (NSCLC) cells (188). These mutations induced an elevated migration and proliferation of NSCLC cells, in which the promoter for the glycoprotein fibulin-5 was found to be hypermethylated. Since fibulin-5 is a protein participating in the aggregation and stabilization of complexes in the extracellular matrix, one may speculate that 2HG can also promote cancerogenesis by optimizing the extracellular milieu for tumor growth.

Renal cell carcinomas were recently found to have a decreased expression of s-2HG-dehydrogenase, which may contribute to carcinogenesis in the respective cells. MDH2 was the main source of s-2HG (147). Similar to other cancers, the inhibition of glutaminase decreased s-2HG production. Thus, the worsened prognosis for renal cell carcinoma patients should be based on a lower expression of s-2HG-dehydrogenase and increased levels of s-2HG.

### 2HG in prevention of immunosurveillance

#### Immune system within the tumor microenvironment

**FIG. 6.**
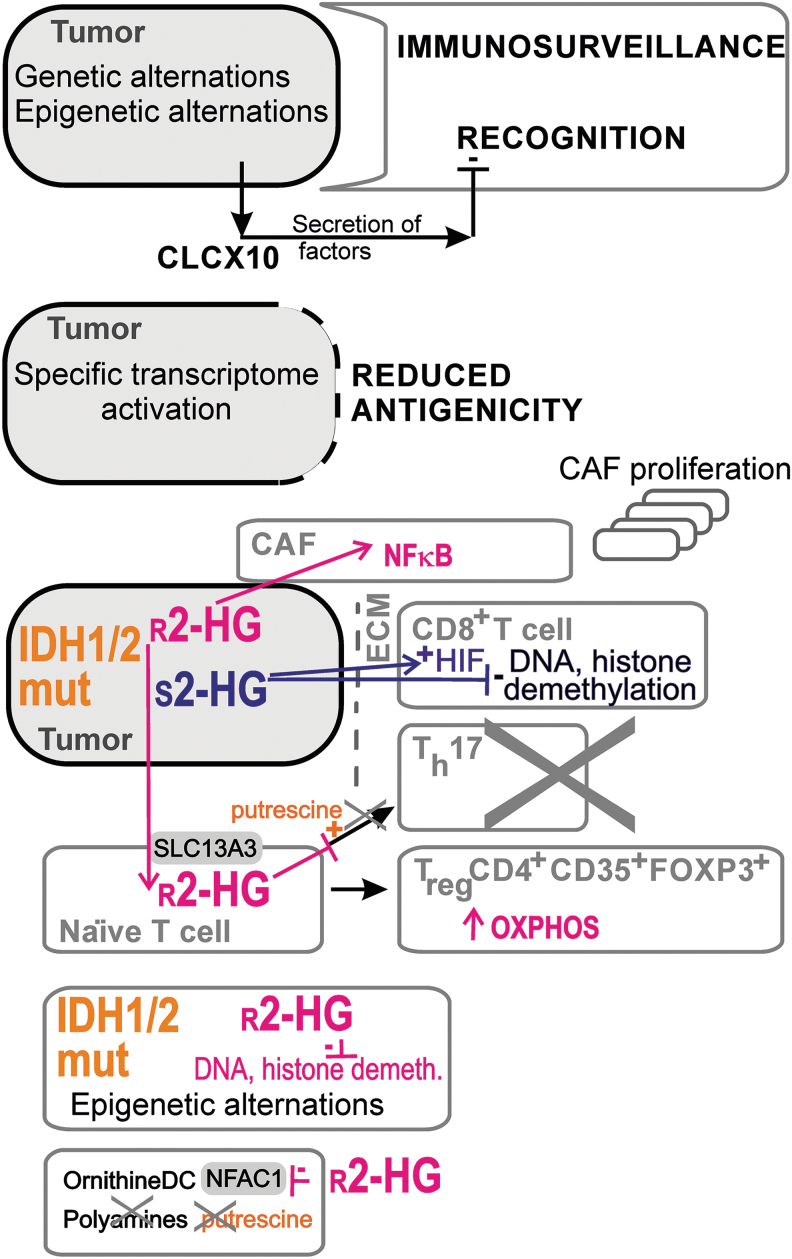
**2HG effects related to immune cells within the tumor microenvironment.** The scheme summarizes the major effects of 2HG within the tumor microenvironment: prevention of tumor recognition (*top*): r-2HG inhibits the secretion of certain factors such as CXCL10. In this way, r-2HG prevents the recruitment of T cells and hence tumor recognition (84, 101). Reduction of antigenicity (*second from the top*): can reset epigenetic and gene expression changes promoted by 2HG. Promoting proliferation of CAF (*third from the top*). Impairment of immune cells and their differentiation (remaining schemes): such as the promotion of Treg cells and the simultaneous blockage of differentiation into the helper T_h_17 cells from the naive T cells; plus effects on HIF transcriptome reprogramming and epigenetics alternations, as was described for tumor cells. *Bottom*: an example of the blockage of polyamine expression due to r-2HG inhibition of the NFATC1 (18). CAF, cancer-associated fibroblasts; CXCL10, C-X-C motif chemokine ligand 10; NFATC1, nuclear factor of activated T cells 1; T_h_, T helper.

Recently, a great deal of attention has been paid to immune system cross talk with tumors and metastases (29, 176). Indeed, cancerogenesis progresses not only due to genetic and epigenetic somatic alternations (34) but also due to failed immunosurveillance, at least to some extent (56) (Fig. 6). The following defects can be identified: (i) Impairment of immune cells, leading to the inability of the immune system to recognize cancer cells or cells in premalignant states. (ii) Active secretion by cancer cells of factors causing the above effects as in (i). These factors can act either systematically or locally within the tumor microenvironment. Interestingly, both 2HG enantiomers have been recently found to fulfill such roles. (iii) The transformation of cancer cells or cells in premalignant states so that they expose their cell surface in a manner reducing antigenicity or to be shielded against adjuvancy.

#### r-2HG effects

Both 2HG enantiomers prevent the immunosuppression of tumors. Thus, several detailed effects of r-2HG that prevent the immunosuppression of tumors were identified. The transport of r-2HG into T cells is facilitated by the sodium-dependent dicarboxylate transporter SLC13A3, irrespective of whether they are activated or naive, and in general impairs their effector function and proliferation (18). r-2HG interferes with the activation of the nuclear factor of activated T cells 1 (NFATC1), a key transcription factor (18). Interestingly, this effect is linked to ATP deficiency since it was rescued by a cell-permeable analog of ATP. r-2HG also acts at the biochemical level in T cells inhibiting ornithine decarboxylase (18). As a result, the biosynthesis of polyamines such as putrescine is hampered. This represents a self-perpetuating effect since putrescin antagonizes r-2HG, suppressing its proliferation.

r-2HG was also found to inhibit the expression of CD12 in dendritic cells and inhibits the secretion of C-X-C motif chemokine ligand 10 (CXCL10), hence preventing the recruitment of T cells (84, 101). In naive T cells, r-2HG destabilizes HIF-1α. Thus, while preserving OXPHOS, r-2HG increases differentiation into the CD4^+^, CD25^+^, FOXP3^+^ line of the so-called T_reg_ cells. This proceeds at the expense of differentiation into T_17_ helper cells (13).

Finally, nonmalignant cells are affected by r-2HG within the tumor microenvironment, such as cancer-associated fibroblasts and myeloid cells. As a result, such a microenvironment is permissible for tumor progression. For example, r-2HG *via* the stimulation of NF-κB elevates proliferation in a stromal niche for AML cells (26) and at relatively small concentrations promotes fibroblast proliferation (43).

#### s-2HG effects

Activated mouse CD8^+^ T cells are affected by s-2HG so that their proliferation and effector function is abrogated due to the resulting inhibition of DNA and histone demethylation and possible activation of HIF (172).

### 2HG as a possible metabolic marker of cancer

#### Normal *versus* pathological levels of r-2HG and s-2HG

**FIG. 7.**
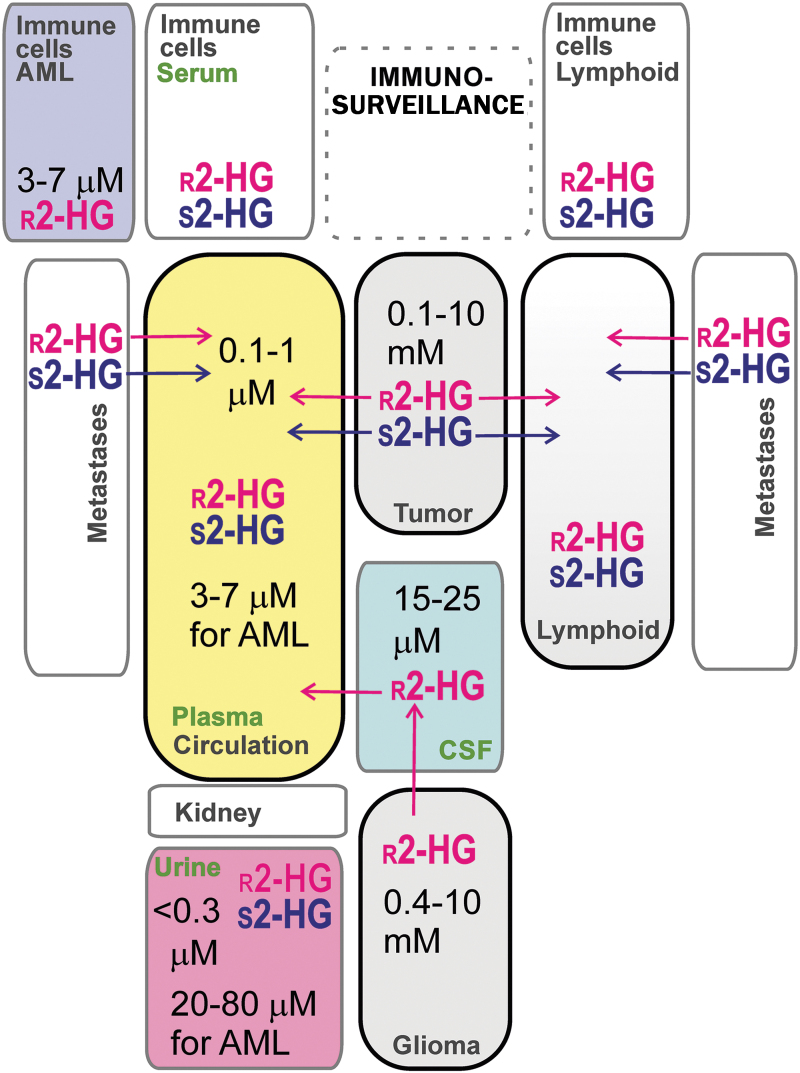
**Possible distribution of 2HG enantiomers in body fluids.** The scheme summarizes exemplar concentrations of 2HG enantiomers analyzed in serum, urine, and immune and cancer cells/tissues (for References, see Table 1 and the text).

In the above sections, it was recognized that the range of r-2HG concentrations in cells and/or tissues can span several orders of magnitude. Theoretically, any analyte with such a large span should be used as a marker of related changes. Thus, in several cases, thresholds were identified for disease-indicating r-2HG concentrations in glioma or AML cells. Similarly, a diagnostic goal is to identify such thresholds in body fluids available for diagnostics (Fig. 7 and Table 1). In a few cases, such thresholds were determined in urine and serum.

Intermediate concentrations, that is, 50–100 times lower than those found in grade II/III gliomas, were found in ER− breast carcinoma cells, HTB-126/Hs 578T, and epithelial adenocarcinoma MDA-MB-231,cells (155). The leakage of r-2HG into body fluids should occur when higher 2HG levels exist in tumors and metastases of numerous cancer types. Table 1 summarizes the reported amounts converted to concentrations.

Focusing on breast cancer, c-Myc-retransformed breast cancer tissues contained substantial levels of 2HG (0.5–20 nmol/mg), despite the absence of IDH1/2 mutations (168). These tumor tissues exhibited global epigenome changes associated with poor prognosis (168). A patient with hormone-receptor (HR+) breast carcinoma exhibited mutant IDH1 R132L within the tumor tissue and cells in lymph nodes, which corresponded to an elevated 2HG concentration in urine (22 ng/mL *vs*. 1.7 ng/mL in healthy controls) and serum (1979 ng/mL *vs*. 105 ng/mL in healthy controls) (50). This is comparable to urine 2HG concentrations for gliomas with IDH1/2 mutations (1–14.6 ng/mL), which were found to be much higher than for gliomas with wt IDH1/2 (1–4 ng/mL) (49). Also, serum 2HG concentrations were in a similar range for gliomas with IDH1/2 mutations (33–283 ng/mL), and these were no different from those for patients with gliomas with wt IDH1/2 (35–277 ng/mL) (49).

Also, elevated s-2HG levels were reported in glioblastoma, pediatric glioblastoma, neuroblastoma, and renal cell carcinoma, besides those found in hypoxic cells (68). Distinguishing between 2HG enantiomers may provide even more correlating diagnostics.

#### Immune cells as sources of r-2HG

Tumor development proceeds in a complex host–tissue microenvironment, in which immune cells play significant pleiotropic roles alongside fibroblasts, the extracellular matrix, and lymphatic vascular networks (157). A distinct type of CD4^+^ T cells producing interleukin (IL)-17, designated as T helper (T_h_) 17 cells (105), was found to switch from OXPHOS to aerobic glycolysis with concomitantly elevated levels of r-2HG and hypermethylated DNA at the locus of *Foxp3* (185). As a result, this locus becomes repressed. Since this drives differentiation of the induced regulatory T cells (iTreg cells) from naive CD4^+^ T cells, such differentiation was hindered. Interestingly, the enhanced metabolic flux through the aspartate aminotransferase GOT1, converting glutamate to 2OG, was responsible for the increased r-2HG (185). One may speculate that the elevated r-2HG can diffuse into the tumor microenvironment and it might be found subsequently in plasma, urine as plasma ultrafiltrate, or lymphatic fluid.

#### Immune cells as sources of s-2HG

Specifically, due to the activation of the immune system, s-2HG could be found in body fluids since certain activated immune cells increase s-2HG within the microenvironment of tumors and metastases. CD8^+^ T cells undergo a switch to aerobic glycolysis mediated by HIF-1α upon migration to hypoxic tumor environments or inflamed tissue. This is accompanied by a profoundly high elevation of s-2HG levels, almost up to 1.5 m*M* already at normoxia, but was dependent on HIF (172). Again, speculatively, this phenomenon may increase plasma levels of s-2HG.

## Future Perspectives

The normal 2HG metabolism and metabolism of related compounds, such as s-2-hydroxyglutaramate, should be definitively studied in noncancer cells to establish a “background” for progressed oncogenesis. Interference of 2HG with HIF and NRF2 systems should be further elucidated. At early stages of oncogenesis and in the absence of IDH1/2 mutations, the effects of “intermediate levels” of both 2HG enantiomers within the 10–100 μ*M* range and their consequences should be further uncovered and determined. Cases and circumstances should be studied in which 2HG could serve as a marker of prognosis, remission, recurrence, or early diagnosis.

2HG's effects on the as yet unidentified particular promoters of genes should be further determined. Promoters of tumor-suppressive miRNA should be investigated with a specific emphasis. They are often large and contain CpG islands to be sensitive to hypermethylation. Currently, unidentified information signaling pathways affected by 2HG enantiomers should be discovered. Uncovering details of 2HG metabolism and signaling in various immune cell types will help to predict cancer recurrence even after tumor excision. All such future knowledge may lead to the establishment of precise diagnostics and/or individual prognoses based on s-2HG and r-2HG as markers of oncogenesis.

## Conclusions

Both 2HG enantiomers, s-2HG and r-2HG, are related to specific cancer metabolism in numerous cancer types. Oxidative stress accompanies their formation, unless other mechanisms stimulate the enhanced expression of antioxidant systems. 2HG accelerates oncogenesis *via* its effects on epigenetics and on cross talk with the immune system. Since 2HG cellular concentrations several orders of magnitudes, they may be employed in the future as metabolic markers when analyzed in relevant body fluids.

## Abbreviations Used

2HG: 2-hydroxyglutarate, both enantiomers
2OG: 2-oxoglutarate (α-ketoglutarate)
2OGDH: 2-oxoglutarate dehydrogenase
ADHFE1: alcohol dehydrogenase iron-dependent isoform 1
Akt: protein kinase B
AML: acute myeloid leukemia
BCAA: branched-chain amino acids
BCAT1,2: BCAA aminotransferases 1,2
CSF: cerebrospinal fluid
DEPTOR: DEP domain-containing mTOR-interacting protein
ER: estrogen receptor
ERK: extracellular-related kinase
FOXO: forkhead box protein O
G6PDH: glucose-6-phosphate dehydrogenase
GDH: glutamate dehydrogenase
GSH: reduced glutathione
H_2_O_2_: hydrogen peroxide
HIF: hypoxia-inducible factor
HOT: hydroxyacid-oxoacid transhydrogenase
IC: isocitrate
IDH1,2,3: isocitrate dehydrogenase isoform 1,2,3
JmjC: Jumonji histone lysine demethylase
JNK: c-Jun N-terminal kinase
KDM: lysine demethylase
LDH: lactate dehydrogenase
LIPT1: lipoyl transferase 1
MAPK: p38 mitogen-activated protein kinase
MAPKK: MAPK kinase
MDH: malate dehydrogenase
ME: malic enzyme
miRNA: microRNA
MLKL: mixed lineage kinase domain-like
MR: magnetic resonance
mtDNA: mitochondrial DNA
mTOR: mammalian target of rapamycin
NF-κB: nuclear factor kappa-light-chain-enhancer of activated B cells
NOX: NADPH oxidase
NRF2: nuclear factor erythroid 2-related factor
NSCLC: non-small-cell lung cancer
OXPHOS: oxidative phosphorylation
PARP: poly(ADP-ribose) polymerase
PDH: pyruvate dehydrogenase
PHD: proline hydroxylase domain enzyme (EglN)
PHGDH: phosphoglycerate dehydrogenase
PLOD: procollagen-lysine 2-oxoglutarate 5-dioxygenase
PPP: pentose phosphate pathway
r-2HG: 2-hydroxyglutarate r-enantiomer, that is, d-2-hydroxyglutarate
RIP: receptor-interacting protein
ROS: reactive oxygen species
s-2HG: 2-hydroxyglutarate s-enantiomer, that is, l-2-hydroxyglutarate
SIRT: sirtuin
SOD: superoxide dismutase
TERT: telomerase reverse transcriptase
TET: ten-eleven translocation methylcytosine dioxygenase
TNFα: tumor necrosis factor-α
wt: wild type
